# Suppurative Pericementitis

**Published:** 1904-04-15

**Authors:** A. B. Conklin


### SUPPURATIVE PERICEMENTITIS.

BY A. B. CONKLIN, M.D.

My only excuse for taking up the consideration of a subject that would seem so clearly to belong to the domain of the stomatologist is that dentists are so often refering those patients to the family physician for treatment, where their oral symptoms are but the local manifestation of a constitutional condition, and the subject is now considered by dental pathologists but another display of that hydra-headed sport of ancestral gout, lithæmia.

While there is no good reason why a dentist may not as properly and ethically advise a consititutional therapeutic treatment to his own patient as to apply the locally therapeutic and prosthetic treatment, yet if he chooses to refer his patient to us, it is none the less incumbent upon us to meet the trust thus reposed with the best service that a thorough knowledge of the subject offers.

We should profit by clearly defining our position in the use of the terms pyorrhœa alveolaris and suppurative pericementitis, which latter term I have chosen to use as being preferable to the term phagedenic pericementitis, suggested by Dr. G. V. Black, since it more accurately conforms to the actual condition present, which is one of purulent liquefaction of tissue, instead of a slough, as the word phagedenic would imply.

Pyorrhœa means simply a flow of pus, while the word alveolaris refers to the alveolus, or alveolar tissue, and the combined use of the words conveys no more definite information than a discharge of pus from the alveolar tissues. Such a pyorrhœa might obtain in the course of various morbid conditions as mercurial and other forms of salivation, phosphorus poisoning, abscess in connection with a devitalized tooth, gangrenous and ulcerative stomatitis and from the encroachment of tartar.

It is the latter condition only that need be given any consideration at this time, as it is the only one named that has, by a portion of the dental profession at least, been included under the name pyorrhœa alveolaris, and is the only one likely to be confounded with a primary pericementitis.

The pyorrhœa, which is the remote result of a primary deposit of tartar about the neck of a tooth, is the terminal stage of a marginal gingivitis which has resulted from the mechanical irritation of the salivary incrustation, and from beginning to end is a purely local condition.

To still apply the term "pyorrhœa" to this condition is to perpetuate an error, since it only applies to the single terminal symptom, and conveys no knowledge of the real disease itself. The term might, with even a greater show of reason, be applied to salivation or alveolar abscess, since the alveolar tissues are in these conditions at least primarily involved. In such cases of salivary calculus, with resulting gingivitis, as show an involvement of the pericemental membrane, it is only after the gums have so far receded as to lay it bare, or inflammation has extended to it through continuity, or pus extending along its surface has carried infection to its deeper parts.

An attempt as been made of late, as a result of a restudy of buccal conditions, to differentiate kindred conditions and restrict the use of the term "pyorrhœa alveolaris" to those cases showing a primary involvement of the pericemental membrane. This is a step in the right direction, for clearer conceptions in pathology point the way to improved methods of treatment and suggest a more fitting nosology.

Pyorrhœa alveolaris, though its use be still permitted, can not have more than a generic application, and by the process of evolution must soon give way to other terms that will more accurately reflect our conceptions of the various species of suppurative inflammations in and about the alveolar tissue. One result of latter-day researches in oral pathology is already apparent in the use of such nosological terms as calcic pericementitis, hematogenic calcic pericementitis, phagedenic pericementitis, suppurative pericementitis, and gouty pericementitus to express a specific affection of the pericemental membrane.

While this enveloping membrane is undoubtedly many times affected secondarily, from a primary marginal gingivitis, it seems now also fairly well established that we may have a primary inflammation of this tissue, in the absence of a pathological condition in any adjacent organ—an idiopathic pericementitis.

I would not include under this classification those forms of pericementitis resulting from the irritation of hypertrophied cementum, carious dentine or devitalized pulp.

No less than fifteen years ago William Henry Potter, of Boston, taught that diseases of the pericemental membrane might be due entirely to constitutional disturbances, which Tomes, even earlier than that, enumerates syphilis and rheumatism as systemic states that may react upon the pericemental membrane.

I wish particularly to call your attention to rheumatism, or the so-called uric acid diathesis, as an etiological factor in pericemental affections.

I shall not take your time to go into the chemistry of uric acid, or the various theories of metabolism that have been brought forward within the last five years to show that it is some member of the xanthin group of nitrogenous waste products, other than uric acid, whose retention in the system gives rise to the complex of symptoms known as lithæmia. It is quite sufficient for our purpose, at this time, to know that such a diathetic state exists and is now recognized as a common cause of the various forms of rheumatism and irregular gout. The alveolo-cemental membrane is at once periosteum and joint tissue, and we know that an arthritis, which may affect any joint in the body, has long been viewed as the single, truly typical expression of the uric acid diathesis.

Joint tissue is largely a dense, fibrous tissue which implies a less active circulation, though its vascular supply, which is largely capillary, may be extensive, and, as Garrod pointed out years ago, fibrous and cartilaginous tissues possess a relatively lower alkalinity than other tissues, and more often contain appreciable quantities of uric acid and urate salts. These are some of the factors which render the alveola-cemental articulation prone to the same conditions that are recognized in other joints, and vulnerable to the same causes. The pericemental membrane lines the socket of the tooth, while covering the alveolar process upon the one side and enclosing the tooth upon its inner surface. Being thus walled in on either side by bony tissue, the chances of accommodating itself to an inflammatory condition, it will be seen, are reduced to a minimum.

The profession both of medicine and dentistry are under lasting obligation to Prof. E. N. Peirce, of Philadelphia, for his painstaking investigations, a few years ago, into the pathology of pericemental affections. The deposit found upon the roots of teeth thus affected was carefully removed and submitted to Prof. Brubaker and Prof. Congdon, of the Drexel Institute for chemical analysis. The hydrochloric acid, destructive distillation and murexide tests were each employed, and with the uniform result of showing the deposits to be composed of either free uric acid crystals, urates, or allied nitrogenous compounds.

Such thorough work by men of unquestioned ability not only accurately shows the composition of these calcic deposits, but by the very fact of establishing their identity, proves their gouty origin. Serumal calculi properly expresses the condition, as does also the term hemato-calcic calculi, the term coined by Prof. Peirce, since the concretions are deposits of salts from the blood, as are gouty deposits found in other joints and elsewhere throughout the body.

The inference being that all of Prof. Peirce's cases had progressed to the stage of pus formation, and its discharge into the mouth, the question has been asked by some of the ultraconservative if these deposits of inorganic salts may not have occured after the development of the pyorrhæa and found ingress through the fistulous opening. To say nothing of the difficulty of accounting for the formation of the gouty salts, from the buccal secretions, we may mention the observations of Dr. J. Lambert Asay, of San Jose, California, which seem to prove the primary nature of the deposit. He has gone farther than Prof. Peirce, by examining the roots of sound teeth imbedded in healthy gums, pain about the root being the only symptom, first cutting through the gums and then removing a portion of the alveolar process, and found during and even before the stage of hyperæmia the same uratic concretions as were found in Prof. Peirce's cases after the pyorrhœa had resulted.

It would seem, therefore, that we must accept the gouty deposit as the very first pathological departure, so far as the tissues of the mouth are concerned, in suppurative pericementitis, and that the term "gouty pericementitis" becomes a most appropriate one.

As pointed out by Dr. Asay, a gouty pericementitis may or may not terminate in pyorrhœa. It is a question of the degree of inflammation and the damage that results to the circulation and the nutrition of the membrane.

Gouty pericementitis is very properly divided into the acute and chronic forms, as we have two distinct types of development. In the milder cases of an acute pericementitis the patient complains of twinges of pain about the roots of the teeth, and along the course of the superior or inferior dental nerve, according to which jaw is affected, and the pain may even extend to other branches of the trifacial nerve. The suffering may be more or less severe while it lasts, is distinctly neuralgic in character, and after a period of variable duration disappears to return again whenever another period of imperfect elimination permits of the accumulation in the system of the nitrogenous waste products.

Since these cases have been found by Dr. Asay to be accompanied by the deposit of uratic granules in and about the pericemental membrane, although as yet an inflammation is not apparent, we may very logically argue that, the pain is due to the pressure upon the terminal nerve filaments from hyperæmia which falls short of actual inflammation.

The intensity of the pain is in keeping with the degree of vascular engorgement, and whenever the irritation from the serumal concretions is such as to produce a greater degree of hyperæmia than the limited opportunities of the membrane for swelling can accommodate, stasis follows, and in hyperæmia with stasis we have inflammation.

Pressure now becomes extreme, swelling occurring in the only direction which it can take, lifts the tooth above its fellows and pain is intense. The inflammation rapidly extends to the gums, which at first are fiery red, but soon take on the dark, purplish discoloration indicative of coagulation necrosis. Pus forms and either discharges directly through the gums, or, by forcing a separation of the membrane from the cementum, escapes at the gingival margin. Such is acute gouty pericementitis, ending in suppuration—suppurative pericementitis—so-called pyorrhœa alveolaris.

In the chronic form of the affection the structural changes which ensue are of very gradual development, and the actual pain experienced is very little, or none at all.

We are all familiar with this form of the disease, as witnessed in those persons where sound teeth imbedded in healthy gums gradually loosen from an absorption of all the tissues about their roots until they actually fall out. The process is to be explained by the pathology of bone softening and absorption as seen elsewhere in the body. A periostitis invites an exudation upon the inner surface of the membrane, which induces an amount of pressure that interferes with the nutrition of the bone, and leads to its softening and absorption. With the absorption of the devitalized membrane and alveolar process, the gums shrink away from the teeth, and their support is gone.

Suppurative pericementitis is found more often in rheumatic subjects, as might be expected, than elsewhere, and Dr. Asay tells us he has "more often than otherwise" seen the chronic form associated with chronic rhinitis. This is a significant coincidence, since our rhinologists now view chronic hypertrophic rhinitis as a toxic rhinitis from lithæmia.

The frequent association of divers recognized manifestations of lithæmia with suppurative pericementitis becomes suggestive of more than an accidental relationship, and with the preponderance of evidence being in support of the constitutional and gouty origin of pericementitis, we should not forget this possible relationship, when these patients are referred to us, but should go most thoroughly into their family history for the purpose of arriving at their inherited tendencies, and question the individual carefully with regard to any former or associate manifestation of lithæmia.

I have had but a limited experience in the treatment of suppurative pericementitis, and, since my attention was drawn to the theory of its lithæmic origin, have had an opportunity to treat less than half a dozen cases and observe them long enough to warrant me in drawing any conclusions. The list of diseases which bear a symptomatic relationship to uric acid toxæmia, as already accepted, is quite large, and in the last five cases of suppurative pericementitis I have been surprised to find in them all other unmistakable manifestations or uric acid toxæmia, at one time or another.

Three of my cases had had rheumatism, three had had recurring tonsillitis, while one each had had chronic eczema, hypertrophic rhinitis and migraine. One case had had rheumatism and recurring tonsillitis, another rheumatism and rhinitis, another a chronic eczema and tonsillitis, a fourth had had recurring attacks of tonsillitis for years, and frequent attacks of migraine, while the fifth gave a history of having had rheumatism only.

The element of heredity could be traced in at least two cases.

While five cases, it is true, is a small number from which to draw any conclusion, still they are all such as to support the theory of the disease which I have briefly outlined to you and strengthen its plausibility.

In the treatment of a suppurative pericementitis, physicians are hardly likely to be called upon to treat the local conditions, and the constitutional treatment is the treatment of the underlying lithæmia. We are to employ such means and methods as have been found most expedient in rheumatism and other manifestations of the dyscrasia.

Three cardinal principles should be covered by whatever plan of treatment is decided upon, viz: (1) limit the amount of nitrogenous food consumed, (2) stimulate oxidation and metabolism, and (3) increase elimination, especially through the kidneys. Medicine can probably render us a real service in the accomplishment of our third purpose only, and herein I have no new remedy to offer. The salicylates and alkalies are unquestionably our best renal depurants, and because the salicylates are prone to disturb the stomach, the alkalies are to be preferred for long continued use, and this is necessary in the treatment of pericementitis.

A further point in palatability is gained by having the alkaline salts made effervescent, and for this reason I have been using lately the preparation known as "alkalithia," which contains the largest percentage of alkalies of any preparation of its class with which I am familiar, and consequently gives the most prompt and pleasing results.

By way of further confirmation of our theory, I wish to add in conclusion, that alkalithia has been used in the five cases above mentioned with a curative effect, that seemed permanent in two cases at least, after a lapse of a year and fifteen months respectively.

While the action of the remedy seemed equally favorable in the other three cases, yet, for the reason that I was not able to keep track of them for so long a time, I am unable to state what were the remote results.

If the results of treatment prove anything, they would seem, in this small series of cases, to prove the gouty origin of suppurative pericementitis.

